# Time-dependent analysis of dosage delivery information for patient-controlled analgesia services

**DOI:** 10.1371/journal.pone.0194140

**Published:** 2018-03-15

**Authors:** I-Ting Kuo, Kuang-Yi Chang, De-Fong Juan, Steen J. Hsu, Chia-Tai Chan, Mei-Yung Tsou

**Affiliations:** 1 Department of Biomedical Engineering, National Yang-Ming University, Taipei, Taiwan; 2 Department of Anesthesiology, Taipei Veterans General Hospital, Taipei, Taiwan; 3 Department of Information Management, Minghsin University of Science and Technology, Hsinchu, Taiwan; 4 Faculty of Medicine, National Yang-Ming University, Taipei, Taiwan; International Manganese Institute, FRANCE

## Abstract

Pain relief always plays the essential part of perioperative care and an important role of medical quality improvement. Patient-controlled analgesia (PCA) is a method that allows a patient to self-administer small boluses of analgesic to relieve the subjective pain. PCA logs from the infusion pump consisted of a lot of text messages which record all events during the therapies. The dosage information can be extracted from PCA logs to provide easily understanding features. The analysis of dosage information with time has great help to figure out the variance of a patient’s pain relief condition. To explore the trend of pain relief requirement, we developed a PCA dosage information generator (PCA DIG) to extract meaningful messages from PCA logs during the first 48 hours of therapies. PCA dosage information including consumption, delivery, infusion rate, and the ratio between demand and delivery is presented with corresponding values in 4 successive time frames. Time-dependent statistical analysis demonstrated the trends of analgesia requirements decreased gradually along with time. These findings are compatible with clinical observations and further provide valuable information about the strategy to customize postoperative pain management.

## Introduction

Pain is the most common chief complaint in hospitalized patients[1, 2]. In addition, the postoperative acute pain increases physical and/or mental burden and even hinders the recovery[3, 4]. Pain relief always plays the essential part of perioperative care and important role of medical quality improvement[5]. Well pain management has great help to increase the speed of rehabilitation for postoperative patients[6]. Most importantly, the subjective experience of pain is hard to measure objectively[7, 8]; only the patient who suffers from pain can make certain characteristics of pain such as intensity, symptom, location and frequency[9].

In order to fit the requirements for individual pain relief, patient-controlled analgesia (PCA) service has been widely used in postoperative pain management[10, 11]. PCA is a method that allows a patient to self-administer small boluses of analgesic under anesthetist’s prescription[12, 13]. The control parameters of PCA device such as bolus dose, lockout interval, background infusion, dose limits and loading dose of the programmable infusion pump should be established before use. Loading dose can be given prior to initiate the PCA therapy which provides the initial accumulation of analgesic for patient. A bolus dose will be injected when the patient triggers the handhold button each time, which is the major procedure of pain control. Lockout interval is a short-time safety mechanism that limits the minimum allowable period between bolus doses. Four hours limit is the long-term protecting mechanism restricting the maximum amount of analgesic in this period. Infusion rate is a continuous dose which keeps the patient’s serum drug concentration of analgesic. When patient triggers the handhold button connecting to PCA device, the bolus dose will be injected for pain relief immediately. PCA has been found to provide better pain control, less complications, shorter hospitalization and greater satisfaction with pain management[14–17].

The concentration of analgesic determine the efficiency for pain relief[18]. PCA device should be set up in medicate program with anesthesiologist’s prescription before therapy. A demand event occurs when a patient triggers the handhold button; the PCA program determines whether the demand was allowed deliver a bolus to patient or not, thus the delivery event occurs with valid demand. The ratio between demand and delivery (D/D ratio) reveals the meaningful response from prescription. For an ideal pain management, the D/D ratio should equal to one, meaning that each demand has one corresponding delivery.

PCA log records all events during a PCA therapy which includes initial drug delivery setting, patient’s pain control behaviors, program reconfiguration, system alarm, and statistical results at the end of therapy. It truly describes the patient’s pain management process during this therapy. Despite the fact that at the end of PCA log, it provides statistical results and the last setting of PCA program, the information is still insufficient to represent the efficiency of this therapy. In fact, it is possible to extract dosage information from PCA log to provide additional features for further understanding of the efficiency of PCA therapy.

Patients usually suffer the greatest pain after operation and emergence from general anesthesia. In the ordinary course of recovery, the analgesic requirement changes along with time. Hence, pain should be alleviated as time goes on in general situation. As a result, the analysis of the dosage information over time provides great help to figure out the variance of patient’s pain relief condition. In order to precisely delineate the trend of pain relief and analgesic requirement over time, we developed a PCA dosage information generator (PCA DIG) to extract valuable information from PCA logs and further integrate the extractions into useful indicators to explore the variations in analgesic demand over time during the course of PCA use[19].

## Methods

### Patients

This study conducted with the approval of Institution Review Broad at Taipei Veterans General Hospital (VGHIRB No.: 2015-03-004BC). We collected data on postoperative PCA patients from May 2005 to April 2010. Exclusion criteria included therapy less than 48 hours, format error of PCA log and missing data. Patients, who used intravenous patient-controlled analgesia (IVPCA) or patient-controlled epidural analgesia (PCEA) for post-operative pain relief, were included in this study. When a patient starts a PCA therapy, an infusion pump (Abbott AIM Plus System, Abbott Laboratories, North Chicago, IL, USA) with drug program will be set by anesthesiologist’s prescription for individual’s needs. PCA team members will routinely visit the patients to record the pain and side-effects and evaluate analgesic scores for checking the effect of pain relief. If patients feel too much pain or uncomfortable side-effects of analgesic, they can notify the nurse station to call PCA team members for additional visitations. In each visit, the parameters of the PCA drug program may be changed according to the patient’s condition.

### PCA dosage information generator

**Fig 1:**
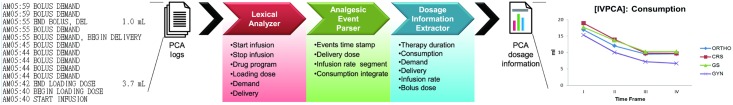
The flowchart of PCA DIG.

PCA log consists of a lot of text messages to record all events during the therapy. The PCA DIG parses PCA log to easily understand dosage information, explores meaningful events and combines information with different time intervals. Due to the duration of one PCA therapy was commonly fallen between 48 and 72 hours, we subsumed the first 48-hour of log and divided the dosage information by 12 hours into 4 time frames (TF). PCA dosage information presented with continuous time frames which included consumption, bolus dose, infusion rate, counts of demand and delivery. PCA DIG consisted of three components (Fig 1): lexical analyzer, analgesic event parser and dosage information extractor. Visual Studio 2008 (Microsoft Corporation, Washington, DC) is utilized to develop PCA DIG.

- Lexical analyzer: PCA log filled with various text messages such as machine model, partition marks, system action, dosage program, power control, alarm, printout, statistical results and so on. Lexical analyzer analyzes and extracts the significant text messages from PCA log which included all the events of power on, power off, date change, infusion start, infusion stop, all dosage programs, demand and delivery, and their corresponding timestamps.
- Analgesic event parser: Dosage program may be changed according to patient’s condition, therefore the order of events becomes very important. This parser parses all the dosage related events with their timestamps from significant text messages and it extracts the dosage delivery capacities and accumulates drug delivery counts. Consumption includes loading dose, bolus dose and calculated infusion rate. While calculating the infusion rate into consumption, the event timestamp and dosage unit must be clearly identified.
- Dosage information extractor: PCA dosage information includes consumption, demand, delivery, infusion rate, and bolus dose. Dosage information is calculated according to the relative time frames. Drug consumption is the sum of the values with loading dose, bolus dose and volume of infusion rate. Demand, delivery and bolus dose are accumulated values. Infusion rate is the average delivery rate in present time frame. The extracted dosage information is used to easily understand values for PCA therapy.

### Statistical analysis

We use the results provided by PCA DIG to discover the relationship between dosage information and time. The statistical data including consumption, demand, D/D ratio, and continuous infusion are showed with relative time frames during the first 48 hours therapy. The 1^st^ to the 12^th^, the 13^th^ to the 24^th^, the 25^th^ to the 36^th^, and the 37^th^ to the 48^th^ hour of therapy are represented with TF_I_, TF_II_, TF_II_ and TF_IV_ respectively. Consumption represents total analgesic delivered to a patient that includes loading dose, bolus dose, and volume of continuous infusion. In PCA therapy, patient triggering the handhold button to obtain a bolus dose delivery is the primitive response. Demand counts represent the pain relief requirement of patient during PCA therapy. D/D ration responds the situation of pain management. If each demand triggers a bolus delivery and patient feels pain relief, the D/D ratio should be equal to 1. High D/D ratio may indicate that this dosage program does not provide enough effect pain relief. Most of PCA dosage program set a continuous infusion rate to maintain a stable concentration of analgesic for patient. It may be regulated many times by the PCA team members according to patient’s condition. In general situation, the continuous infusion rate should be regulated to a lower rate for patient with a stable and well pain management. In order to explore the rate changing trend of continuous infusion over time, we compare the rates of two neighboring time frames and define the comparison results as following three groups: positive (P), zero (Z), and negative (N). Positive represents that the patient needs higher continuous infusion rate than that of the previous time frame. Zero represents that the patient uses the same continuous infusion during this time frame. Negative represents that the patient needs lower continuous infusion during this time frame. Further, we compare the infusion rate of each time frame to the initial (Ini) infusion rate. All PCA dosage information is expressed as mean ± SD. We summarize it by medical department to fit with the same kind of similar operation. Two-way analysis of variance (2-way ANOVA) was used to compare the difference in infusion rate between each time interval and its predecessor. A *p* value less than 0.05 was considered statistically significant. All statistical analyses are conducted with Microsoft Excel 2010 (Microsoft Corporation, Washington, DC) and SPSS Statistics 24.0 (IBM Corp. Armonk, NY).

## Results

We adopted the retrospective analysis and included the medical departments with PCA cases above 500 for an objective statistic. IVPCA therapy included departments of orthopedics (ORTHO), colon rectal surgery (CRS), general surgery (GS) and gynecology (GYN). PCEA included departments of orthopedics, general surgery and chest surgery (CS). Demographic data with all cases are shown in Tables 1 and 2. The average age of ORTHO, CRS and GS are over 60 years old. The average BMI of ORTHO is higher than other medical departments.

**Table 1:** Demographic data with 4852 cases for IVPCA therapy.

Department	ORTHO		CRS		GS		GYN	
	Number (%)	Mean ± SD	Number (%)	Mean ± SD	Number (%)	Mean ± SD	Number (%)	Mean ± SD
**Cases**	2323 (47.9%)		1082 (22.3%)		775 (16.0%)		672 (13.8%)	
**Gender**								
**Male**	1021 (44.0%)		616 (56.9%)		421 (54.3%)		0 (0%)	
**Female**	1302 (56.0%)		466 (43.1%)		354 (45.7%)		672 (100.0%)	
**Age (year)**		61.6 ± 18.2		65.5 ± 13.8		60.8 ± 16.4		48.6 ± 12.4
**Weight (kg)**		64.1 ± 13.2		61.8 ± 11.5		61.4 ± 12.6		58.8 ± 11.2
**Height (cm)**		158.7 ± 10.4		160.5 ± 8.4		160.8 ± 8.5		157.0 ± 5.7
**BMI (kg/m**^**2**^**)**		25.8 ± 14.2		23.9 ± 3.7		23.7 ± 4.1		23.8 ± 4.3

**Table 2:** Demographic data with 3652 cases for PECA therapy.

Department	ORTHO		GS		CS	
	Number (%)	Mean ± SD	Number (%)	Mean ± SD	Number (%)	Mean ± SD
**Cases**	1614 (44.2%)		1037 (28.4%)		1001 (27.4%)	
**Gender**						
**Male**	568 (35.2%)		701 (67.6%)		624 (62.3%)	
**Female**	1046 (64.8%)		336 (32.4%)		377 (37.7%)	
**Age (year)**		69.5 ± 12.8		63.1 ± 14.9		58.0 ± 16.8
**Weight (kg)**		65.5 ± 11.8		62.8 ± 11.7		61.8 ± 11.0
**Height (cm)**		156.6 ± 8.7		162.8 ± 8.4		162.8 ± 8.4
**BMI (kg/m**^**2**^**)**		26.7 ± 4.5		23.7 ± 3.7		23.3 ± 3.7

The statistical results of IVPCA dosage information with different medical departments are shown in Table 3. Consumption decreases from TF_I_ to TF_III_ and becomes resembling values from TF_III_ to TF_IV_ in ORTHO, CRS, and GS. Demand obviously decreases from TF_I_ to TF_III_ in all medical departments and becomes a little higher from TF_III_ to TF_IV_ in ORTHO, CRS, and GS. D/D ratio decreases from TF_I_ to TF_III_ in ORTHO and CRS; decreases from TF_I_ to TF_IV_ in GS and GYN. Infusion rate becomes lower starting from initial setting to TF_IV_ among all medical departments.

**Table 3:** Statistical results of dosage information for IVPCA cases.

	TF	ORTHO	CRS	GS	GYN
**Consumption** **(ml)**	I	16.97 ± 7.90	18.69 ± 9.92	17.86 ± 9.54	15.42 ± 6.35
	II	12.13 ± 7.68	13.81 ± 8.50	13.94 ± 8.71	10.17 ± 5.51
	III	9.58 ± 6.15	9.75 ± 6.40	10.54 ± 7.72	7.33 ± 4.38
	IV	9.68 ± 6.36	9.60 ± 6.26	10.63 ± 7.95	6.89 ± 4.39
**Demand** **(times)**	I	25.88 ± 36.71	31.66 ± 40.76	27.23 ± 36.17	21.39 ± 27.14
	II	12.57 ± 17.24	18.02 ± 20.32	18.59 ± 22.43	11.06 ± 17.35
	III	8.64 ± 13.00	9.84 ± 13.33	11.20 ± 16.52	5.98 ± 9.91
	IV	9.59 ± 13.91	10.44 ± 13.24	11.66 ± 16.05	5.07 ± 8.52
**D/D ratio**	I	2.79 ± 3.04	3.12 ± 3.04	2.61 ± 2.32	2.13 ± 1.44
	II	1.65 ± 1.16	2.03 ± 1.35	1.91 ± 1.26	1.45 ± 0.94
	III	1.62 ± 1.32	1.79 ± 1.70	1.83 ± 2.44	1.39 ± 0.84
	IV	1.66 ± 1.30	1.87 ± 2.54	1.78 ± 1.30	1.33 ± 0.59
**Infusion rate** **(ml/hr)**	Initial	0.62 ± 0.33	0.62 ± 0.34	0.63 ± 0.42	0.55 ± 0.16
	I	0.60 ± 0.33	0.60 ± 0.34	0.62 ± 0.43	0.52 ± 0.16
	II	0.53 ± 0.31	0.53 ± 0.33	0.56 ± 0.42	0.45 ± 0.17
	III	0.47 ± 0.30	0.46 ± 0.31	0.50 ± 0.42	0.39 ± 0.18
	IV	0.46 ± 0.29	0.45 ± 0.31	0.49 ± 0.42	0.38 ± 0.18

The statistical results of PECA dosage information with different medical department are shown in Table 4. Consumption has similar values between TF_I_ to TF_II_ and TF_III_ to TF_IV_ but decreases from TF_II_ to TF_III_ among all medical departments. Time variances of demand values in all medical departments are different. Demand decreases from TF_I_ to TF_III_ and becomes a little higher from TF_III_ to TF_IV_ in ORTHO; keeps decreasing from TF_I_ to TF_IV_ in GS, and increases a little from TF_I_ to TF_II_ and TF_III_ to TF_IV_ but decreases from TF_II_ to TF_III_ in CS. D/D ratio keeps decreasing from TF_I_ to TF_IV_ in ORTHO and CS, decreases from TF_I_ to TF_III_ and becomes a little higher from TF_III_ to TF_IV_ in GS. Infusion rate becomes lower starting from initial setting to TF_IV_ among all medical departments.

**Table 4:** Statistical results of dosage information for PCEA cases.

	TF	ORTHO	GS	CS
**Consumption** **(ml)**	I	55.35 ± 18.38	70.88 ± 20.77	71.17 ± 15.45
	II	54.59 ± 20.21	71.63 ± 22.64	71.51 ± 17.12
	III	47.81 ± 18.41	66.56 ± 21.90	67.07 ± 18.27
	IV	48.30 ± 18.71	66.26 ± 21.58	67.78 ± 18.83
**Demand** **(times)**	I	18.94 ± 29.92	19.48 ± 29.07	16.16 ± 27.94
	II	15.43 ± 22.23	17.83 ± 21.28	16.90 ± 25.73
	III	10.49 ± 17.23	12.79 ± 19.58	11.13 ± 17.92
	IV	11.68 ± 17.91	12.71 ± 16.61	11.75 ± 17.41
**D/D ratio**	I	2.73 ± 3.39	2.86 ± 4.31	2.62 ± 2.99
	II	2.25 ± 2.04	2.15 ± 2.27	2.40 ± 2.09
	III	2.26 ± 2.48	1.88 ± 1.60	2.08 ± 1.63
	IV	2.31 ± 2.37	2.01 ± 4.39	2.04 ± 1.55
**Infusion rate** **(ml/hr)**	Initial	3.64 ± 0.89	4.71 ± 1.13	4.90 ± 0.81
	I	3.53 ± 0.98	4.70 ± 1.24	4.89 ± 0.86
	II	3.39 ± 1.09	4.55 ± 1.26	4.82 ± 0.95
	III	3.20 ± 1.13	4.41 ± 1.40	4.71 ± 1.03
	IV	3.18 ± 1.15	4.39 ± 1.34	4.69 ± 1.06

**Fig 2:**
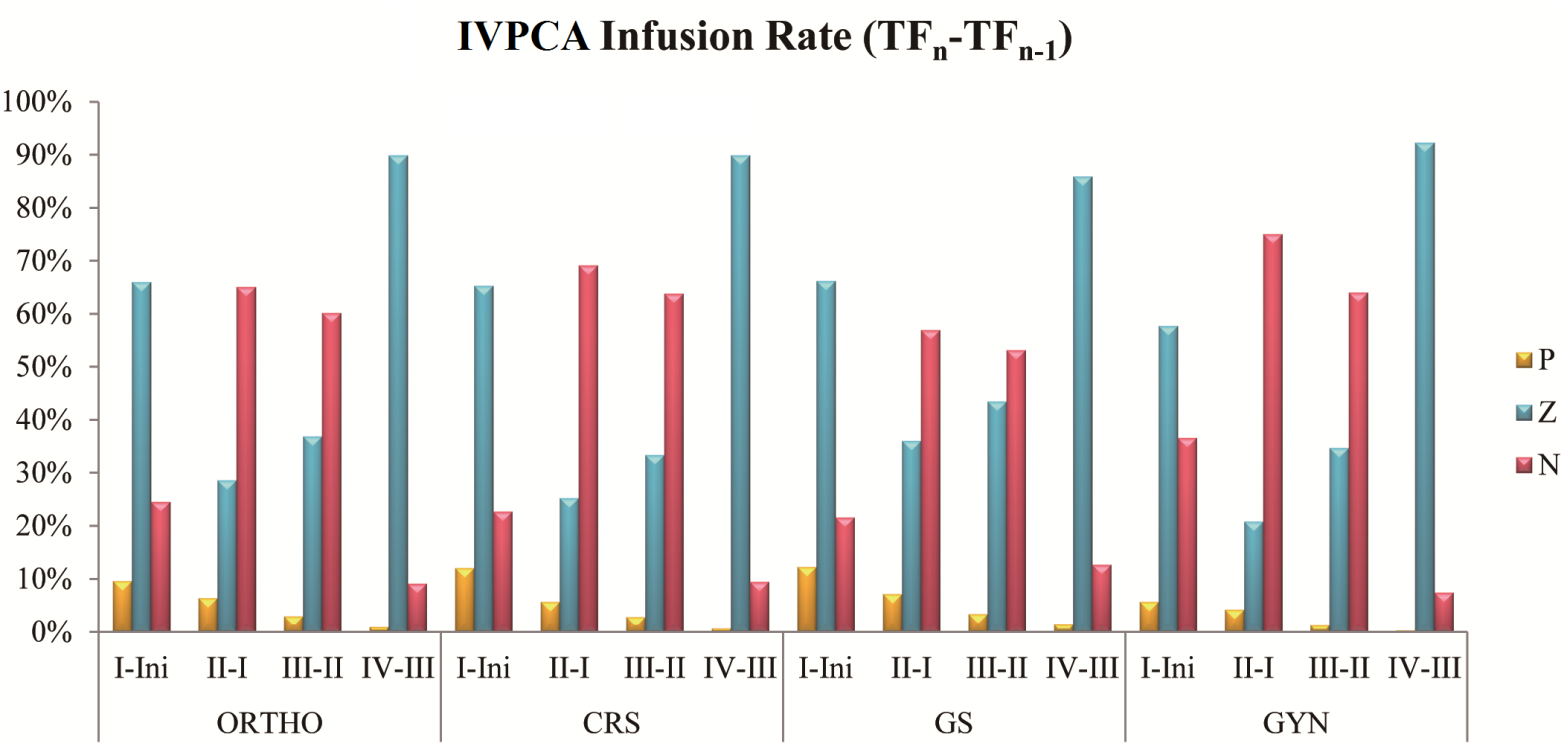
Percentages of the continuous infusion rate changing trend from previous time frame to current frame in IVPCA cases. P, Z, and N represented increased infusion, no change, and decreased infusion respectively (see Methods).

The percentages of the continuous infusion rate changing trend from previous time frame to current frame in IVPCA cases are shown in Fig 2. From TF_I_ to TF_IV_, group P were 9.6%, 6.4%, 2.9%, and 0.9%; group Z were 65.9%, 28.6%, 36.9%, and 89.9%; group N were 24.5%, 65.0%, 60.2%, and 9.1% in ORTHO respectively. From TF_I_ to TF_IV_, group P were 12.0%, 5.6%, 2.8%, and 0.7%; group Z were 65.3%, 25.2%, 33.4%, and 89.9%; group N were 22.7%, 69.1%, 63.8%, and 9.5% in CRS respectively. From TF_I_ to TF_IV_, group P were 12.3%, 7.1%, 3.4%, and 1.3%; group Z were 66.2%, 36.0%, 43.5%, and 86.1%; group N were 21.5%, 56.9%, 53.2%, and 12.6% in GS respectively. From TF_I_ to TF_IV_, group P were 5.7%, 4.2%, 1.3%, and 0.3%; group Z were 57.7%, 20.8%, 34.7%, and 92.3%; group N were 36.6%, 75.0%, 64.0%, and 7.4% in GYN respectively. In “TF_I_—Initial”, 57.7% to 66.2% cases adopt the same setting, 21.5% to 36.6% cases need lower dose and 5.7% to 12.3% cases need higher dose. In “TF_II_—TF_I_”, 56.9% to 75.0% cases need to reduce dose, 20.8% to 36.0% cases adopt the same setting and 4.2% to 7.1% cases need higher dose. In “TF_III_—TF_II_”, 53.2% to 64.0% cases need to reduce dose, 33.4% to 43.5% cases adopt the same setting and 1.3% to 3.4% cases need higher dose. In “TF_IV_—TF_III_”, 86.1% to 92.3% cases adopt the same setting, 7.4% to 12.6% cases need to reduce dose and 0.3% to 1.3% cases need higher dose.

**Fig 3:**
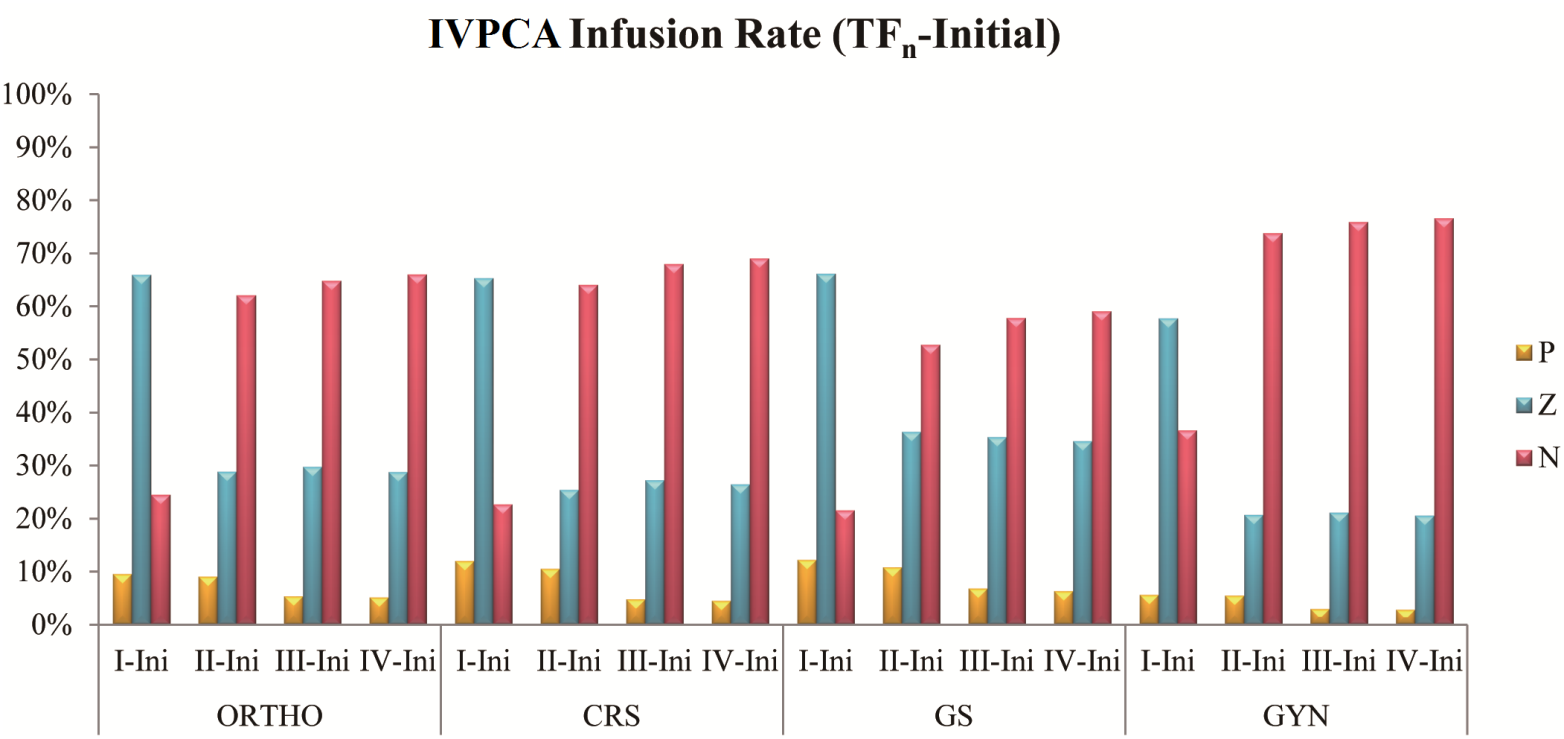
Percentages of the continuous infusion rate changing trend from initial time to current frame in IVPCA cases. P, Z, and N represented increased infusion, no change, and decreased infusion respectively (see Methods).

The percentages of the continuous infusion rate changing trend from initial time to the current frame in IVPCA cases are shown in Fig 3. From TF_I_ to TF_IV_, group P were 9.6%, 9.0%, 5.4%, and 5.2%; group Z were 65.9%, 28.8%, 29.8%, and 28.8%; group N were 24.5%, 62.1%, 64.8%, and 66.0% in ORTHO respectively. From TF_I_ to TF_IV_, group P were 12.0%, 10.5%, 4.8%, and 4.5%; group Z were 65.3%, 25.4%, 27.2%, and 26.5%; group N were 22.7%, 64.1%, 68.0%, and 69.0% in CRS respectively. From TF_I_ to TF_IV_, group P were 12.3%, 10.8%, 6.8%, and 6.3%; group Z were 66.2%, 36.4%, 35.4%, and 34.6%; group N were 21.5%, 52.8%, 57.8%, and 59.1% in GS respectively. From TF_I_ to TF_IV_, group P were 5.7%, 5.5%, 3.0%, and 2.8%; group Z were 57.7%, 20.7%, 21.1%, and 20.5%; group N were 36.6%, 73.8%, 75.9%, and 76.6% in GYN respectively. In TF_I_, 57.7% to 66.2% cases adopt the same setting as initial setting, 21.5% to 36.6% cases need lower dose than initial setting and 5.7% to 12.3% cases need higher dose than initial setting. In TF_II_, 52.8% to 73.8% cases need lower dose than initial setting, 20.7% to 36.4% cases adopt the same setting as initial setting and 5.5% to 10.8% cases need higher dose than initial setting. In TF_III_, 57.8% to 75.9% cases need lower dose than initial setting, 21.1% to 35.4% cases adopt the same setting as initial setting and 3.0% to 6.8% cases need higher dose than initial setting. In TF_IV_, 59.1% to 76.6% cases need lower dose than initial setting, 20.5% to 34.6% cases adopt the same setting as initial setting and 2.8% to 6.3% cases need higher dose than initial setting.

**Fig 4:**
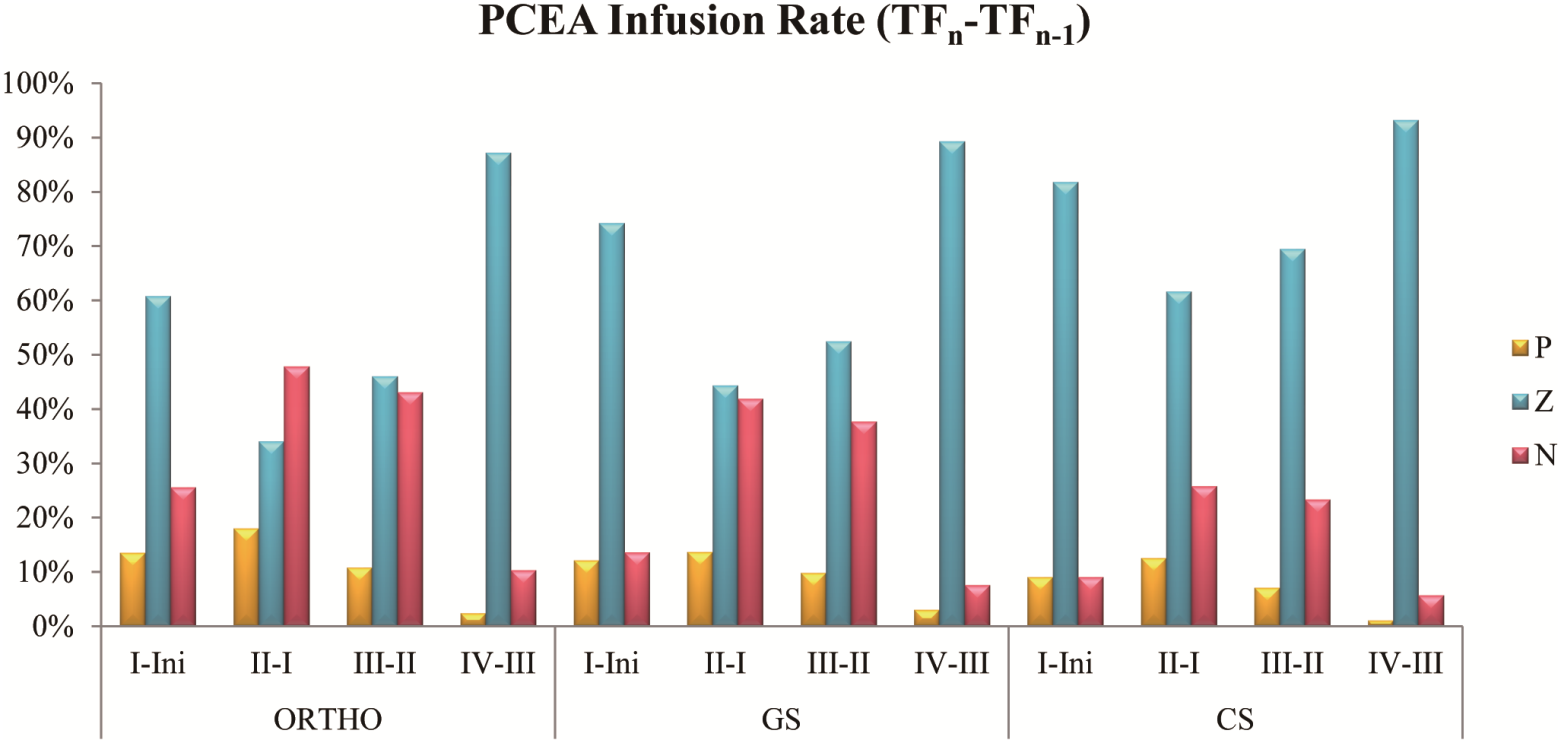
Percentages of the continuous infusion rate changing trend from previous time frame to current frame in PCEA cases. P, Z, and N represented increased infusion, no change, and decreased infusion respectively (see Methods).

The percentages of the continuous infusion rate changing trend from previous time frame to current frame in PCEA cases are shown in Fig 4. From TF_I_ to TF_IV_, group P were 13.6%, 18.0%, 10.8%, and 2.4%; group Z were 60.8%, 34.1%, 46.0%, and 87.2%; group N were 25.6%, 47.9%, 43.1%, and 10.3% in ORTHO respectively. From TF_I_ to TF_IV_, group P were 12.2%, 13.7%, 9.8%, and 3.1%; group Z were 74.3%, 44.4%, 52.5%, and 89.3%; group N were 13.6%, 41.9%, 37.7%, and 7.6% in GS respectively. From TF_I_ to TF_IV_, group P were 9.1%, 12.6%, 7.1%, and 1.1%; group Z were 81.8%, 61.6%, 69.5%, and 93.2%; group N were 9.1%, 25.8%, 23.4%, and 5.7% in CS respectively. In “TF_I_—Initial”, 60.8% to 81.8% cases adopt the same setting, 9.1% to 25.6% cases need lower dose and 9.1% to 13.6% cases need higher dose. In “TF_II_—TF_I_”, 34.1% to 61.6% cases adopt the same setting, 25.8% to 47.9% cases need to reduce dose and 12.6% to 18.0% cases need higher dose. In “TF_III_—TF_II_”, 46.0% to 69.5% cases adopt the same setting, 23.4% to 43.1% cases need to reduce dose and 7.1% to 10.8% cases need higher dose. In “TF_IV_—TF_III_”, 87.2% to 93.2% cases adopt the same setting, 5.7% to 10.3% cases need to reduce dose and 1.1% to 3.1% cases need higher dose.

**Fig 5:**
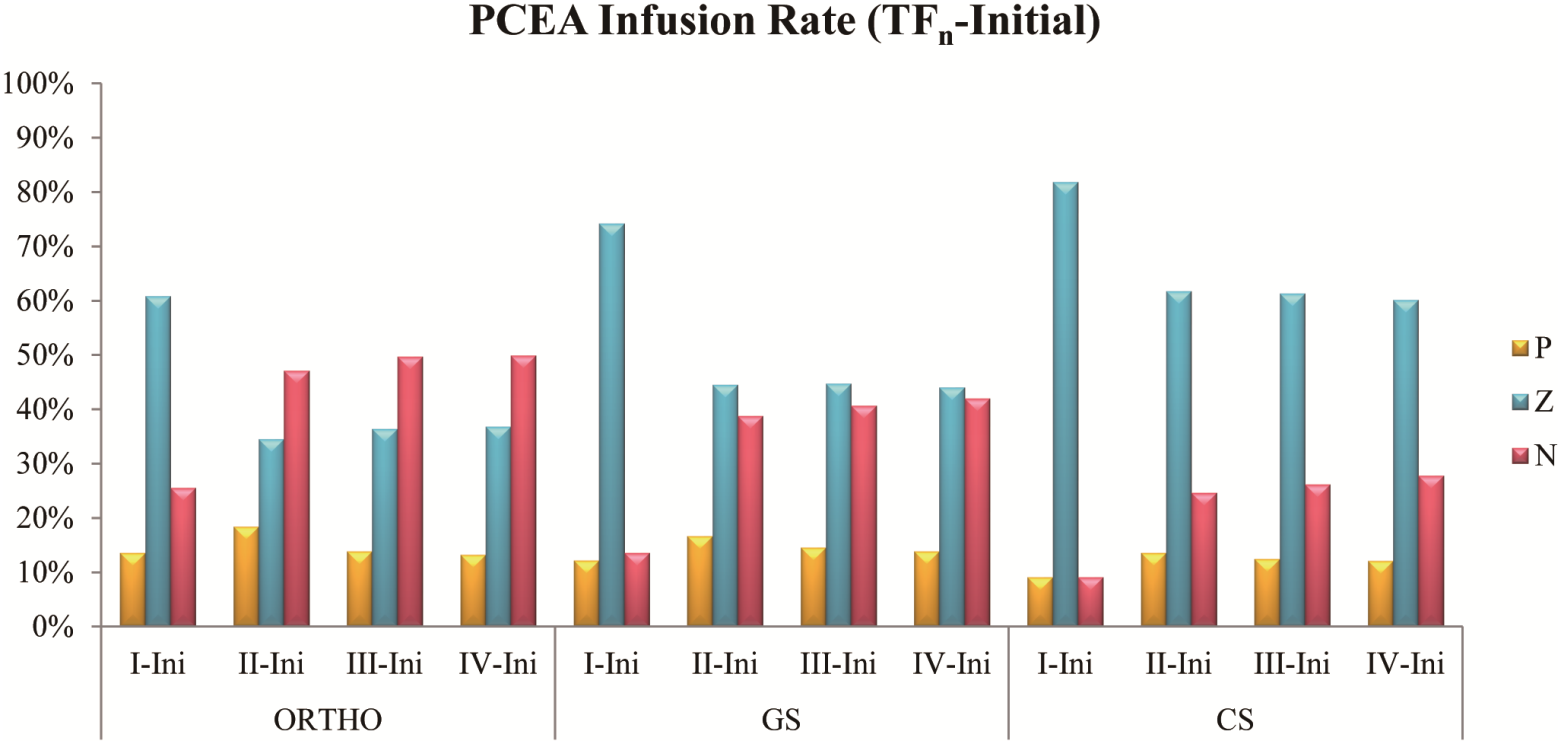
Percentages of the continuous infusion rate changing trend from initial time to current frame in PCEA cases. P, Z, and N represented increased infusion, no change, and decreased infusion respectively (see Methods).

The percentages of the continuous infusion rate changing trend from initial time to current frame in PCEA cases are shown in Fig 5. From TF_I_ to TF_IV_, group P were 13.6%, 18.4%, 13.9%, and 13.3%; group Z were 60.8%, 34.5%, 36.4%, and 36.8%; group N were 25.6%, 47.1%, 49.7%, and 49.9% in ORTHO respectively. From TF_I_ to TF_IV_, group P were 12.2%, 16.7%, 14.6%, and 13.9%; group Z were 74.3%, 44.6%, 44.7%, and 44.1%; group N were 13.6%, 38.8%, 40.7%, and 42.0% in GS respectively. From TF_I_ to TF_IV_, group P were 9.1%, 13.6%, 12.5%, and 12.1%; group Z were 81.8%, 61.7%, 61.3%, and 60.1%; group N were 9.1%, 24.7%, 26.2%, and 27.8% in CS respectively. In TF_I_, 60.8% to 81.8% cases adopt the same setting as initial setting, 9.1% to 25.6% cases need lower dose than initial setting and 9.1% to 13.6% cases need higher dose than initial setting. In TF_II_, 34.5% to 61.7% cases adopt the same setting as initial setting, 24.7% to 47.1% cases need lower dose than initial setting and 13.6% to 18.4% cases need higher dose than initial setting. In TF_III_, 36.4% to 61.3% cases adopt the same setting as initial setting, 26.2% to 49.7% cases need lower dose than initial setting and 12.5% to 14.6% cases need higher dose than initial setting. In TF_IV_, 36.8% to 60.1% cases adopt the same setting as initial setting, 27.8% to 49.9% cases need lower dose than initial setting and 12.1% to 13.9% cases need higher dose than initial setting.

**Fig 6:**
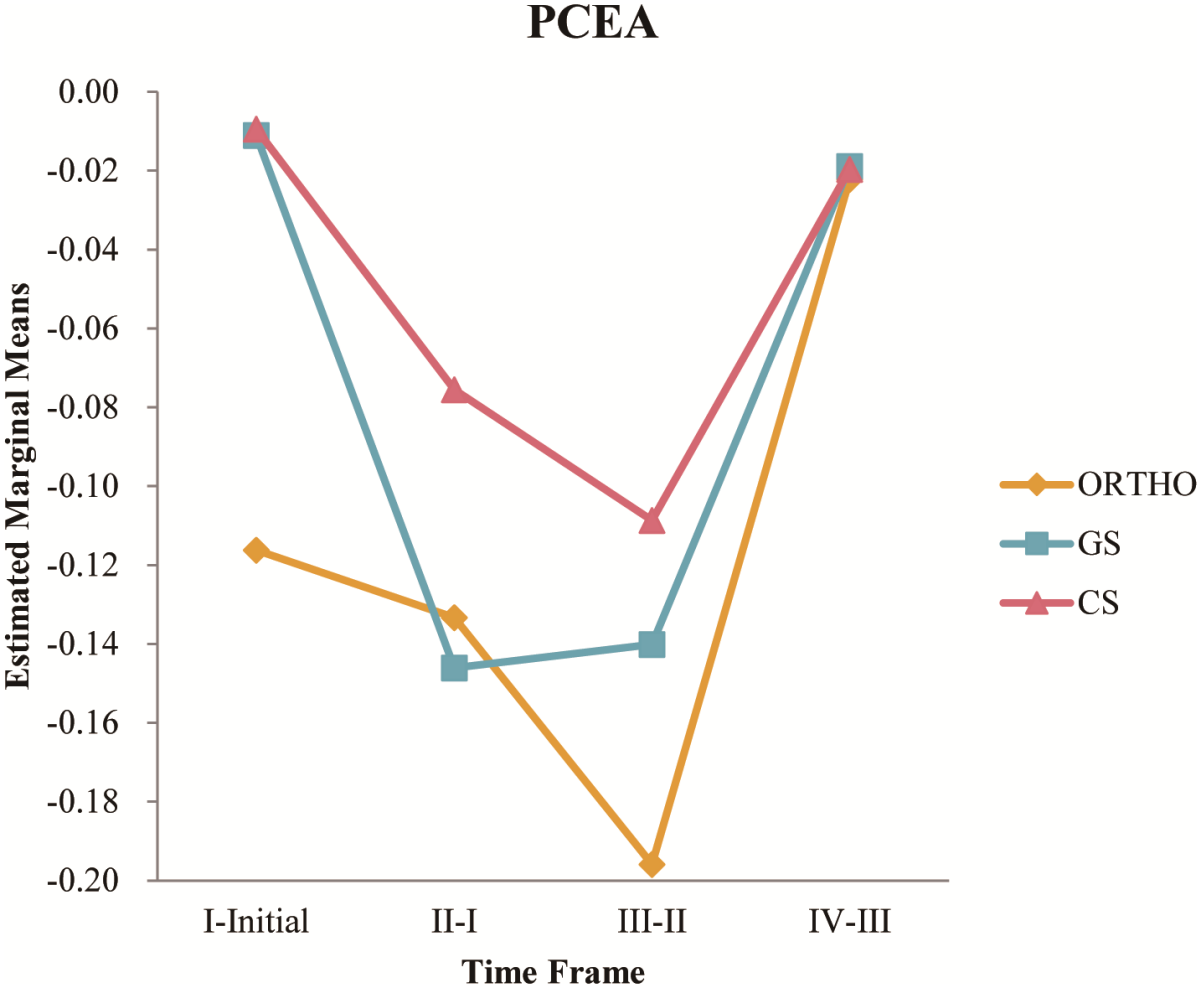
Estimated marginal means of value.

The results of 2-way ANOVA are presented as Table 5. Note that there was no interaction effect between department and timeframe on the difference in infusion rate at distinct time points for patients using IVPCA. The department effect was not significant either but the timeframe effect on difference in infusion rate was significant. The differences in infusion rate of the four timeframes were -0.02, -0.065, -0.063 and -0.008, respectively. In contrast, all the department, timeframe and interaction effect were significant for those using PCEA and the results are illustrated as Fig 6.

**Table 5:** Analytical results of 2-way ANOVA.

Source	IVPCA			PCEA		
	*df*	F	*p*	*df*	F	*p*
**Department**	3	1.969	.116	3	23.719	.000
**Timeframe**	3	93.691	.000	3	56.664	.000
**Interaction**	9	.903	.521	9	5.723	.000

*df*: degrees of freedom

## Discussion

We developed a dosage information generator to provide an easy way to understand features from PCA logs during the first 48 hours of a therapy. PCA dosage information including consumption, demand, delivery, infusion rate, and bolus dose is presented with corresponding values in 4 successive time frames. The statistical results of PCA dosage information show the patients’ pain management conditions over time. The trends of analgesic requirement are decreased gradually over time during PCA therapy[20]. It reflects that self-recovery of human body may affect the analgesia requirement. Our dosage information generator offers valuable information which is essential for further exploring the change in analgesic demand over time in the clinical setting. It also gives clinicians a guide to adjust their PCA prescription based on more solid evidence, instead of less reliable clinical experience.

The statistical results on IVPCA are shown in Tables 1 and 3, Figs 2 and 3. Consumption becomes similar from TF_III_ to TF_IV_ representing patients of ORTHO, CRS and GS are in stable conditions after the first day. The stable condition may happen after 48 hours with GYN patients. To compare the consumption with different medical departments, dosages of GYN are all lower than others. We assume that gender, lighter weight and smaller wounds may affect analgesic consumption[21, 22]. Due to the reason that there are only female patients, the weight and damages of operation are lighter than other medical departments. In ORTHO, CRS and GS, demand of TF_IV_ is higher than TF_III_ that may be the cause of stable patient need to rehabilitate in the second day in order to speed up recovery. The wounds may be pulled during the activities to increase analgesic requirements. Serious pain usually cause higher D/D ratio; the highest D/D ratio appears at TF_I_ and it becomes sable from TF_II_ to TF_IV_ among all medical departments. These results show that patients feel most pain during the first 12 hour after surgery, because patients usually wake up from general anesthesia with analgesic not maintaining an effective concentration. Most of the patients get a well pain management after the first 12 hour of therapy with the average D/D ratio less than 2. The continuous infusion in all medical departments has similar variances with time. Most patients adopt the same setting at TF_I_ and TF_IV_, adjust to lower dosage at TF_II_ and TF_III_. These results show that most patients’ conditions become stable from TF_II_ to TF_III_; patients with lower dosage still maintain well pain management which can verify by D/D ratio within the same time frames. Results of comparing each time frame of continuous infusion with initial setting indicate that most patients need lower dosage than initial setting since TF_II_. It can prove that patients have stable condition at TF_II_ as well. Few patients need higher dosage at TF_IV_ representing that initial setting may not suit for these patients.

The statistical results with PCEA cases are shown in Tables 2 and 4, Figs 4 and 5. Consumption is similar from TF_I_ to TF_II_ and TF_II_ to TF_III_ representing that the variances change by day. The trends of analgesic requirements still decrease along with time[23]. To compare the consumption with different medical departments, dosages of ORTHO are all lower than the others[24]. Because the operation of ORTHO is usually in limbs, but GS and CS is usually in viscera area with malignancies. Big and deeper wounds may cause strong pain and require much dosage setting. In ORTHO, demand keeps decreasing from TF_I_ to TF_III_ but becoming a little higher at TF_IV_. Because these patients need rehabilitation since the second day, the wounds may be pulled during the activities to increase analgesic requirements. Demand of GS becomes stable since the second day represents requirements of analgesia start to decrease. In CS, demand at TF_I_ is lower than TF_II_ being the cause of the operation that usually involved lungs. These patients need sedation to cannulate and assist with respirator until their condition becomes well after surgery. Thus these patients wake up and began to feel the pain at TF_II_ usually. Most patients use the same setting at TF_I_ and TF_IV_, and regulate to lower dosage at TF_II_ and TF_III_. These results show that most patients’ conditions become stable from TF_II_ to TF_III_. They use lower dosage that still keeps a well pain management which can verify by D/D ratio at the same time frames. Results of comparing each time frame of continuous infusion with initial setting indicates that most patients need lower dosage than initial setting since TF_II_ in ORTHO and GS. It can prove that patients have stable condition at TF_II_ also. Few patients need higher dosage at TF_IV_ representing that initial setting may not suit for these patients. In CS, most therapies use the same continuous infusion setting because of the location of operations usually lays on nerve conduction way; these patients need longer resting time.

Serious pain usually causes higher D/D ratio; the highest D/D ratio appears at TF_I_ and becomes sable from TF_II_ to TF_IV_ in all medical departments[25]. This is because patients usually waking up from general anesthesia with analgesic not maintaining an effective concentration. Most of the patients get well pain management after the first 12 hour of therapy. The continuous infusion in all medical departments has similar variances to prove that the analgesia requirements decrease with time. Even the dosage program being set to lower with time, the D/D ratio can still controls within stable area.

In conclusion, PCA dosage information can be the basic database for further research. Our study provided data pre-processing and established data warehouse in data mining process. Through the data mining process, the patient anesthetic condition will be generated as referable medication. Anesthesiologists can make use of the patients’ analgesic information to optimize dosage consumption. It can lead to lower adverse effects, enhance patient safety, promote patients’ satisfaction, and upgrade overall medical quality.

## Supporting information

S1 Table IVPCA data. (XLSX)

S2 Table PCEA data. (XLSX)
